# Controlled Polymerization of Acrylamide via One-Pot and One-Step Aqueous Cu(0)-Mediated Reversible-Deactivation Radical Polymerization

**DOI:** 10.1021/acs.macromol.3c00343

**Published:** 2023-06-19

**Authors:** Zishan Li, Jing Lyu, Yinghao Li, Bei Qiu, Melissa Johnson, Hongyun Tai, Wenxin Wang

**Affiliations:** † Charles Institute of Dermatology, School of Medicine, University College Dublin, Dublin 4 D04 V1W8, Ireland; ‡ School of Chemistry, Bangor University, Deiniol Road, Bangor, Gwynedd LL57 2UW, U.K.; § School of Mechanical and Materials Engineering, University College Dublin, Dublin 4 D04 V1W8, Ireland; ⊥ Aust Hefei Institute for Advanced Research, Anhui University of Science and Technology, Hefei 232001, China

## Abstract

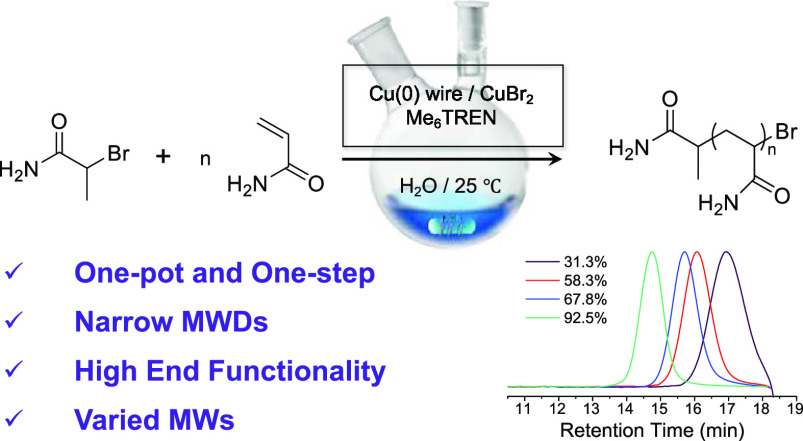

Copper-catalyzed controlled polymerization of acrylamide (AM) has always been a challenge, which typically exhibits low monomer conversion and broad molecular weight distribution (MWD) or requires complex/multistep reaction procedures, due to the highly active nature of the AM radical and its side reactions. To overcome the above challenges, herein, we report the successful synthesis of well-defined polyacrylamide (PAM) via a facile one-pot and one-step aqueous Cu(0)-mediated reversible-deactivation radical polymerization (RDRP). The results of this strategy show that strong deactivation control is the key for the controllability of AM RDRPs, which depends on the equilibria of polymerization and mutual conversion of different copper species. With the fast-propagating monomer AM, extra addition of Cu^II^ into the reaction system is an effective way to enhance deactivation. Based on this kinetically controlled strategy, well-defined PAMs with narrow molecular weight distributions (MWDs) and varied molecular weights (*M*_w_s) were successfully achieved.

## Introduction

Polyacrylamide (PAM), a class of polymers with a wide range of applications such as oil recovery,^1^ soil conditioning,^2,3^ and fillers in surgery,^4^ has been mainly synthesized via free radical polymerization (FRP). In recent years, increasing demand for enhanced control over the molecular wight/distributions (*M*_w_/MWDs) and complex architectures facilitated the employment of reversible-deactivation radical polymerization methods (RDRP) for controlled synthesis of PAM. For example, McCormick et al. achieved controlled polymerization of AM via reversible addition–fragmentation chain transfer (RAFT) polymerization,^5^ using a range of chain transfer agents (CTAs), e.g., 2–1-carboxy-1-methyl-ethylsulfanylthiocarbonylsulfanyl)-2-methyl-propionic acid (CMP), 4-cyanopentanoic acid dithiobenzoate (CTP), etc.^6,7^ Another robust RDRP technique, copper (Cu)-catalyzed RDRP (e.g., atom transfer radical polymerization (ATRP)), has attracted considerable attention in recent years due to its applicability to a broad range of monomers and the ability to synthesize polymeric materials with a well-defined structure, predetermined *M*_w_, and narrow MWDs.^8−10^ However, even though various Cu-catalyzed RDRP approaches for controlled polymerization of AM have also been attempted, they are still problematic. The challenges involved in Cu-catalyzed RDRP of AM typically lie in the low monomer conversions and broad MWDs of the resulted polymers. For example, RDRP of AM catalyzed by CuCl/CuCl_2_/bipyridine complex in water (H_2_O) suffered from the problem of the poorly controlled polymerization process with low *M*_w_ tailing, broad dispersity (*Đ* = ∼1.70), and limited monomer conversion (*Conv*. <38%).^11^ In a later report, significantly narrowed dispersity of PAMs (*Đ* = 1.24–1.25) was achieved by utilizing pentamethyldiethylenetriamine (PMDETA) as the ligand; however, monomer conversion was quite low, which only reached 9%.^12^ A poor control over the *M*_w_s of PAMs synthesized using tetramethylethylenediamine (TMEDA) as a ligand, CuCl as a catalyst, and chloroacetic acid as an initiator was also reported, where although narrower MWDs were obtained (*Đ* = 1.19–1.24), only a low monomer conversion was reached (less than 25% even after 48 h),^13^ and an obvious M W deviation from the theoretical value was observed.^13,14^ The problem of relatively high dispersity (*Đ* > 1.40) was also observed in PAMs prepared under a Cu(I)/tris[2-(dimethylamino)ethyl]amine (Me_6_TREN)-catalyzed RDRP process in aqueous media.^15^ The successful controlled polymerizations of AM via Cu-catalyzed RDRP require complex or multistep reaction procedures.^16−19^ For example, by performing electrochemically mediated ATRP (eATRP) of AM in H_2_O/dimethyl formamide (DMF), PAMs with well-controlled *M*_w_s (consistency between experimental *M*_w_ and theoretical values), and narrow MWDs (*Đ* < 1.2) were obtained.^16^ In a further report, controlled polymerization of AM via a simplified eATRP (seATRP) was carried out in pure H_2_O.^17^ However, both eATRP and seATRP of AM require an extra electrolysis reaction set-up. More recently, controlled polymerizations of AM monomers were achieved via Cu(0)-mediated RDRP with Me_6_TREN as a ligand in H_2_O; however, a two-step reaction procedure and the prior synthesis of a tailor-designed initiator are required.^18,19^ Overall, at present, the Cu-catalyzed RDRPs of AM have been suffering from limitations such as low monomer conversion (low yield), poorly controlled *M*_w_s/MWDs, and complex or multistep reaction procedures.

To solve the above problems, in this work, we report a facile and efficient one-pot and one-step aqueous Cu(0)-mediated RDRP strategy for well-controlled AM polymerization. Through this new approach, the well-controlled polymerizations of AM have been successfully achieved with well-defined PAM structure and a range of *M*_w_s.

## Results and Discussion

To achieve successful Cu-catalyzed polymerization of a particular monomer targeting controlled *M*_w_s and MWDs, appropriate selection of the reaction parameters (e.g., initiator, ligand, solvent, and the amount of deactivator etc.) is crucial.^20^ Before determining the reaction parameters, the different monomer types should be carefully considered. In this work, the challenging problem with the controlled polymerizations of AM lies in the nature of the AM monomer in water. First, AM is a fast-propagating monomer with a high propagation rate constant (*k*_p_) (*k*_p_ = 3.93 × 10^4^ L mol^–1^ s^–1^ at 25 °C^21^); this indicates that a high level of deactivation control is required to be competitive for the control of chain propagation. Second, the primary amide group in the acrylamide structure has the tendency of complexation with Cu^II^ species, which would retard the deactivation step, resulting in compromised control over the radical termination and the number of monomer units that could be added to a propagation center during one active cycle. Moreover, H_2_O is a very polar solvent with a high ATRP equilibrium constant (*K*_ATRP_), making the aqueous ATRP of acrylamide more difficult to control compared to that in organic solvents. Issues such as hydrolysis, elimination of the ω-chain end, etc. in H_2_O are also matters.^22,23^ Therefore, compared to other fast-propagating vinyl monomers, controlling the AM polymerization process is more challenging.

With the above insights, for the aqueous Cu(0)-mediated RDRP of AM, the initiator was first selected based on the consideration of its activity (relative to the monomer), water solubility, and accessibility. Here, a commercially available initiator, 2-bromopropionamide (BPA) was chosen. BPA is water-soluble and has a structure resembling that of AM, and as such, its activity is similar to that of the polymer chain end contributing to the concurrent growth of all polymer chains. Then, in terms of the selection of the ligand, its activity is required to match the monomer’s activity.^24−26^ In general, more active ligands provide better control for monomers with high *k*_p_ (e.g., acrylates etc.), and less active ligands are more suitable for low *k*_p_ monomers (e.g., methacrylates and styrenes etc.). Given the high value of *k*_p_ of AM, a highly active ligand Me_6_TREN was selected.

By choosing the above reaction parameters, BPA as the initiator and Me_6_TREN as the ligand, the Cu(0)-mediated RDRP of AM was first performed with Cu(0) wire as the catalyst in H_2_O at 25 °C with a monomer and initiator ratio ([M]_0_/[I]_0_) of 100/1. However, this polymerization led to broad MWDs (*Đ* > 2.5, entry 2 in Table 1) with obvious tails in the size exclusion chromatography (SEC) traces (Figure S1), indicating that severe chain terminations occurred. This resulted in a limited monomer conversion, only 31.1% was reached in 75 min, and a poor control over the chain growth.

**Table 1:** SEC Analysis of Cu(0)-Mediated RDRP of AM with Cu(0) and Cu(0) and 15% Cu^II^ as the Catalyst

entry	catalyst	time (min)	*M*_n,th_^a^ (kDa)	*M*_n,SEC_^b^ (kDa)	*Đ*^b^	conv.^b^ (%)
1	Cu(0)^c^	75	2.20	183.90	1.71	31.1
2	Cu(0)^c^	120	3.10	108.80	2.57	43.4
3	Cu(0) and 15% Cu^d^	10	2.30	3.04	1.39	32.1
4	Cu(0) and 15% Cu^d^	30	2.90	3.19	1.46	40.8
5	Cu(0) and 15% Cu^d^	120	3.10	3.27	1.49	43.9

a *M*_n,th_ = ([M]_0_/[I]_0_) × monomer conversion (conv.) × *M*_w_ (M).

b Number-average molecular weights (*M*_n,SEC_), dispersity (*Đ*), and conv. were characterized using size exclusion chromatography (SEC) equipped with an RI detector.

c Reaction conditions: [M]:[I]:[L] = 100:1:0.18.

d [M]:[I]:[Cu^II^]:[L] = 100:1:0.15:0.23; M = AM; I = BPA; Cu^0^ = pretreated Cu(0) wire (*l* = 5 cm, *d* = 1 mm); Cu^II^ = CuBr_2_; L = Me_6_TREN; solvent = H_2_O (20 mL); *T* = 25 °C.

**Scheme 1:**
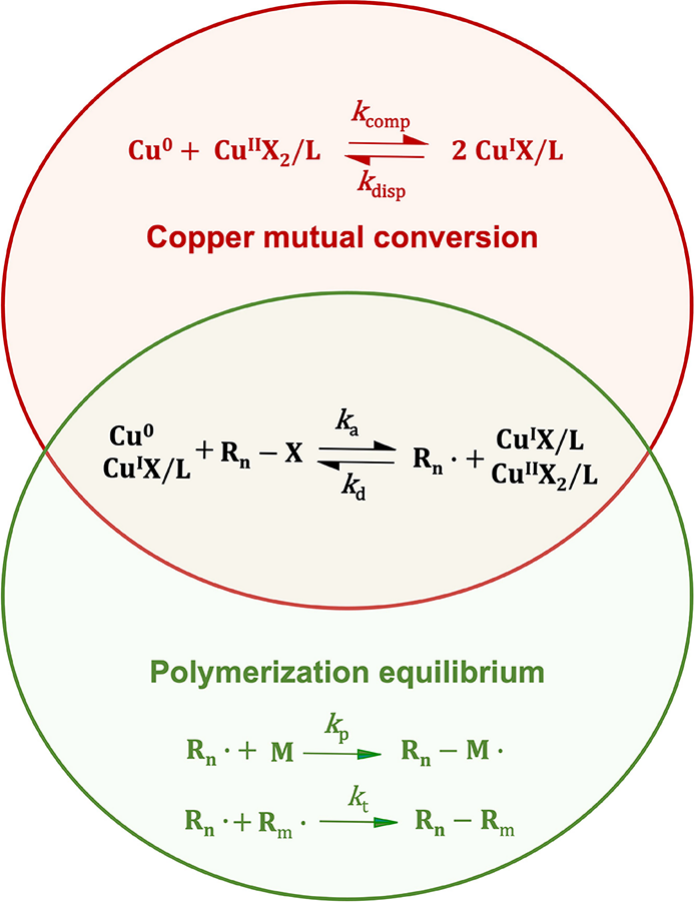
Schematic Representation of the Two Equilibria in Cu(0)-Mediated RDRP Polymerization equilibrium and the copper mutual conversion equilibrium.

To improve the controllability, enhancing the deactivation control by adding Cu^II^ to the reaction system would be the most straightforward way without changing other desired parameters (initiator, ligand, solvent etc.). However, it should be understood that whether this strategy works or not for the Cu(0)-mediated RDRP of AM depends on the relative strength of the two equilibria in the reaction system, polymerization equilibrium (activation/deactivation, propagation, and termination) and the mutual conversion equilibrium of different copper species (Scheme 1). With a slow polymerization equilibrium in nonaqueous solvents, more time would be available for copper mutual conversion, in which case the extra Cu^II^ added would be mostly consumed in the comproportionation process rather than serving as the deactivator for kinetic control.^20^ However, considering that the AM monomer holds a very high *k*_p_, which would lead to a fast polymerization equilibrium; therefore, in the system of Cu(0)-mediated RDRP of AM, the extra added amount of Cu^II^ would quickly participate in the deactivation/activation equilibrium to enhance the deactivation control. In view of that, adding extra deactivator Cu^II^ to the system should be an efficient way to improve its controllability.

Based on the above considerations, maintaining the selected conditions, BPA as the initiator, Me_6_TREN as the ligand, Cu(0) wire as the catalyst, and H_2_O as the solvent, an extra 0.15 equiv Cu^II^ (mol % to initiator) was added at the beginning to investigate its polymerization behavior. As shown in entries 3–5 in Table 1 and Figure S2, indeed, compared to the polymerization catalyzed by Cu(0) wire only, adding 15% Cu^II^ significantly improved the polymerization control, reflected by the diminished SEC tails (i.e., reduced chain terminations, a higher monomer conversion of 40.8% was achieved in 30 min), more uniform SEC traces and narrower MWDs (*Đ* = 1.4–1.5). Nevertheless, due to the unique issues related to the aqueous Cu-catalyzed AM polymerization, i.e., fast chain propagation, potential coordination between the primary amide group with Cu^II^ species and the use of high *K*_ATRP_ solvent etc., obviously, the extra added 0.15 equiv Cu^II^ was still not enough to provide sufficient deactivation control for well-controlled AM polymerization. Thus, subsequently, keeping all other conditions the same, the Cu(0)-mediated RDRP of AM was further carried out by adding 0.4 equiv Cu^II^ to the system.

**Figure 1:**
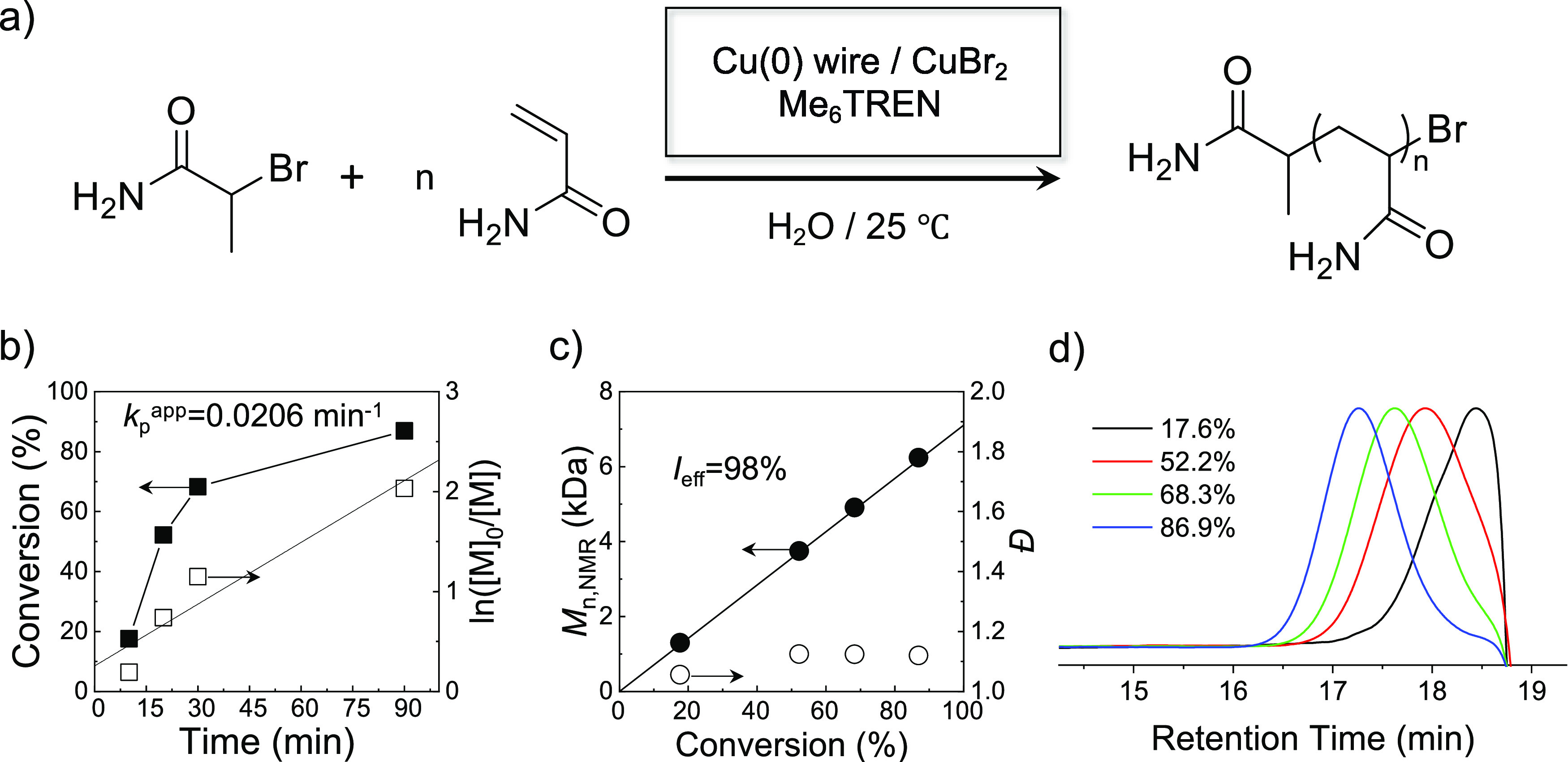
(a) Schematic illustration of the controlled polymerization of AM via one-pot and one-step aqueous Cu(0)-mediated RDRP. (b) Kinetic plots of conversion and ln([M]_0_/[M]) *versus* time, (c) plots of *M*_n,NMR_ and *Đ versus* conversion (the straight line represents the evolution of number-average theoretical *M*_w_s *versus* conversion; *I*_eff_ represents the initiator efficiency, calculated by the ratio of *M*_n,th_/*M*_n,NMR_, Table 2), and (d) the evolution of *M*_w_ monitored by SEC for aqueous Cu(0)-mediated RDRP of AM with [M]_0_/[I]_0_ of 100/1 (b–d), BPA as the initiator, Cu(0) and Cu^II^ as catalysts, and Me_6_TREN as the ligand.

In this case, as expected, a well-controlled polymerization process of AM was successfully achieved with a monomer conversion over 85% in 90 min (Table 2, Figure 1). It is interesting to see that the addition of more Cu^II^ decelerated the polymerization at the initial stage (Conv. = 17.6% in 10 min, Table 2 vs Conv. = 32.1% in 10 min, Table 1) but accelerated the polymerization at the later stage (Conv. = 68.3% in 30 min, Table 2 vs Conv. = 40.8% in 30 min, Table 1). Given that in the pure aqueous system, comproportionation reactions are unfavorable, the extra added Cu^II^ in Table 2 was more favorable to serve as deactivators to participate in polymerization, thus making the polymerization rate slower. While polymerization progressed, the polymerization rate of the system with 40% Cu^II^ added (Conv. = 68.3% in 30 min, Table 2) exceeded that of the system in Table 1 with 15% Cu^II^ added (Conv. = 40.8% in 30 min). This greatly increased monomer conversion within the same reaction period was due to the further improved control over the chain growth and livingness (i.e., greatly diminished chain termination) brought about by the addition of more Cu^II^ as deactivators, this resulted in a slower initial polymerization rate but a continuous growth of all chains during the entire polymerization process. The controlled polymer chain growth can be proved by the parallel evolution of the SEC traces (Figure 1d) and the narrow MWDs (low *Đ* ∼ 1.10) (Figure 1c). The living polymerization nature was clearly verified from the linear increase of number-average *M*_w_s (*M*_n,NMR_) with conversion (Figure 1c) and the good agreement between *M*_n,NMR_ and the theoretical *M*_w_s (*M*_n,th_) (Table 2).

**Table 2:** ^1^H NMR and SEC Analysis of Cu(0)-Mediated RDRP of AM

entry	[M]:[I]:[Cu^II^]:[L]^a^	time (min)	*M*_n,th_^b^ (kDa)	*M*_n,NMR_^c^ (kDa)	*Đ*^d^	conv.^c^ (%)
1	100:1:0.4:0.6	10	1.25	1.30	1.06	17.6
2		20	3.71	3.75	1.13	52.2
3		30	4.85	4.91	1.12	68.3
4		90	6.18	6.24	1.12	86.9

a Reaction conditions: M = AM; I = BPA; Cu(0) = pretreated Cu(0) wire (*l* = 5 cm, *d* = 1 mm); Cu^II^ = CuBr_2_; L = Me_6_TREN; Solvent = H_2_O (20 mL); *T* = 25 °C; [M]_0_ = 1.5 M.

b *M*_n,th_ = ([M]_0_/[I]_0_) × Monomer conversion (Conv.) × *M*_w_ (M).

c Number-average molecular weights (*M*_n,NMR_) and Conv. were determined by ^1^H NMR (Figure S4).

d Dispersity (*Đ*) was characterized using size exclusion chromatography (SEC) equipped with an RI detector.

Moreover, to further confirm the livingness of the polymerization achieved via the above aqueous Cu(0)-mediated RDRP strategy, a chain extension experiment was also carried out by isolating the above-generated polymer at 90 min as the macroinitiator (PAM-Br). The chain extension experiment of the PAM-Br macroinitiator (*M*_n,NMR_ = 6.30 kDa, *Đ* = 1.15, Figures S7, 2) was performed by polymerizing AM monomers with a ratio of [M]_0_/[PAM-Br]_0_ of 200/1, Me_6_TREN as the ligand, and Cu(0) wire and Cu^II^ as catalysts in H_2_O at 60 °C (a higher temperature of 60 °C was adopted here to accelerate the polymerization rate; see detailed purification process and chain extension experimental procedure in the Supporting Information). After a 6-day polymerization period (this much lower polymerization rate is, on the one hand, due to the lower monomer and initiator concentrations in the chain extension experiment −0.55 and 0.0027 M, respectively, on the other hand, might be attributed to the lower activity of the macroinitiator compared to the small molecule one), a conversion of 74.7% was reached, and a clear shift from the PAM-Br to the chain-extended polymer (*M*_n,NMR_ of 16.90 kDa and a narrow dispersity *Đ* of 1.18, Figures S8, 2) was observed from the SEC traces without tailing. Similarly, block copolymerization of the PAM-Br macroinitiator (synthesized under the same recipe as described in Table 2, *M*_n,NMR_ = 5.83 kDa, and *Đ* = 1.15, Figures S9 and S10) and 2-hydroxyethyl acrylamide (HEAA) was performed with a ratio of [HEAA]_0_/[PAM-Br]_0_ of 100/1, with Me_6_TREN as the ligand and Cu(0) wire and Cu^II^ as catalysts in H_2_O at 60 °C (see the detailed block copolymerization procedure in the Supporting Information). As shown in Figure S9, a clear and parallel shift from the macroinitiator, PAM-Br to the block copolymer PAM-b-PHEAA (*M*_n,NMR_ of 15.46 kDa, and *Đ* = 1.19, Figures S9 and S11) was observed in the SEC traces. These outcomes also prove the good livingness of the polymerization process.

**Figure 2:**
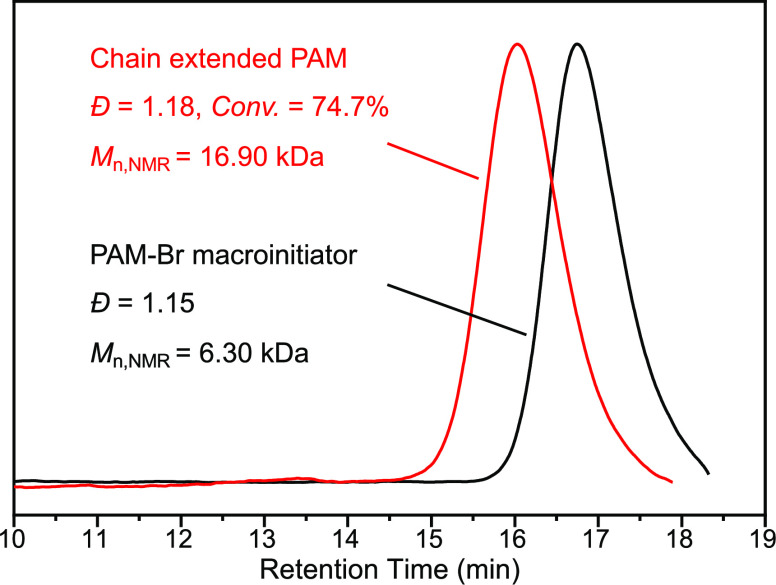
SEC traces of the PAM-Br macroinitiator (*Đ* = 1.15, *M*_n,NMR_ = 6.30 kDa) and chain-extended PAM (*Đ* = 1.18, *M*_n,NMR_ = 16.90 kDa) synthesized via one-pot and one-step aqueous Cu(0)-mediated RDRP.

Previous literature has described eATRP, seATRP (where a complex electrolysis reaction set-up is required),^16,17^ and Cu(0)-mediated RDRP (where a pre-disproportionation step is required prior to charging the deoxygenated monomer and initiator).^18^ However, the above well-defined PAM was generated through one-step successive reactions after charging all the reagents into one reaction flask, i.e., a one-pot and one-step procedure (as described in Figure 1a and detailed experimental procedures in the Supporting Information). These results demonstrate that compared to the previous reported results from ATRP,^11,13,15^ a well-controlled polymerization of AM with low *Đ* and high monomer conversion was achieved via this facile one-pot and one-step aqueous Cu(0)-mediated RDRP strategy, in which the extra addition of Cu^II^ is an effective way to improve the controllability.

**Figure 3:**
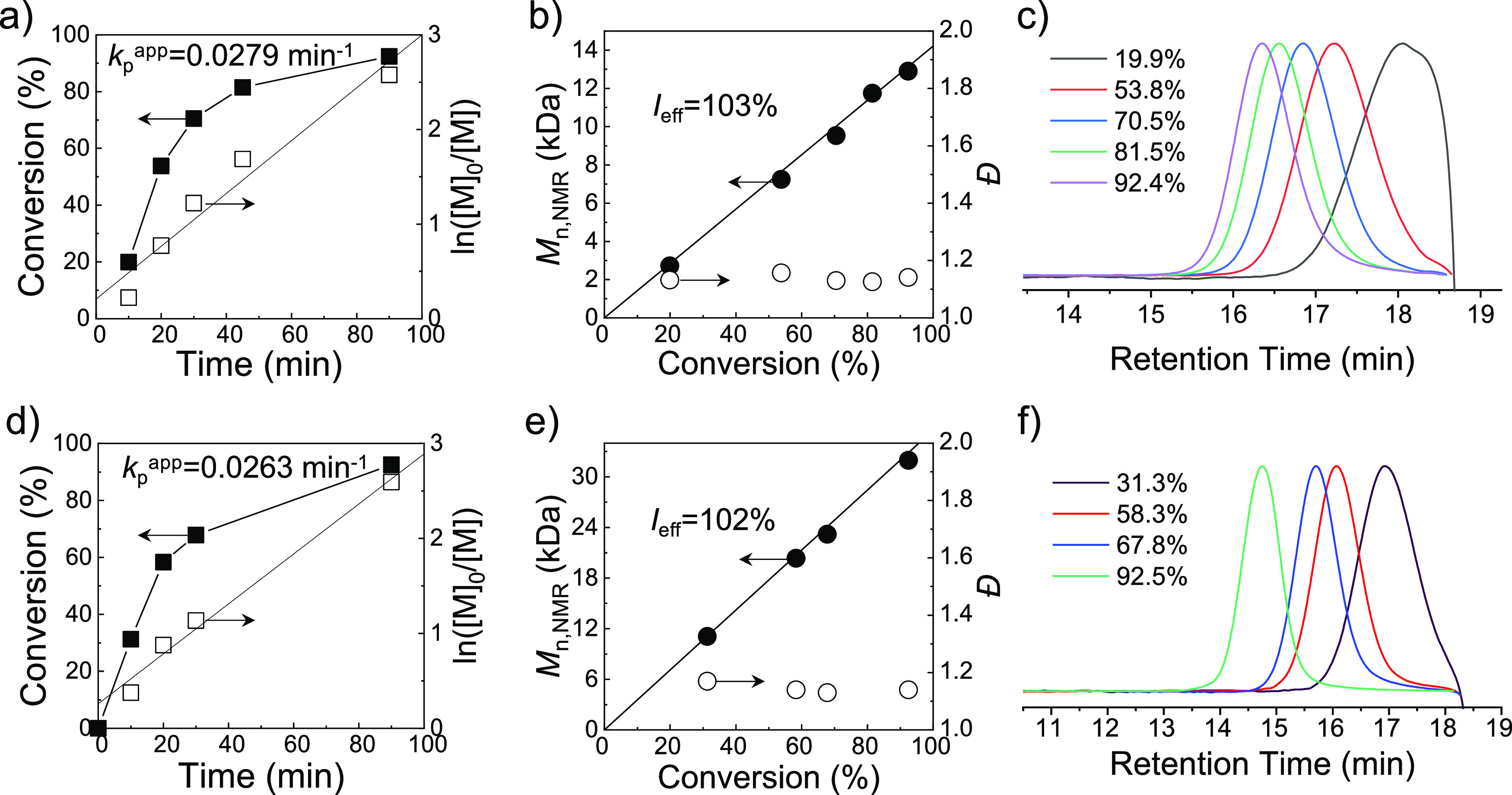
Kinetic plots of conversion and ln([M]_0_/[M]) *versus* time (a and d), plots of *M*_n,NMR_ and *Đ versus* conversion (b and e, the straight line represents the evolution of the number-average theoretical *M*_w_s *versus* conversion; *I*_eff_ represents the initiator efficiency, calculated by the ratio of *M*_n,th_/*M*_n,NMR_, Table 3), and the evolution of *M*_w_ monitored by SEC (c and f) for aqueous Cu(0)-mediated RDRP of AM with [M]_0_/[I]_0_ of 200/1 (a–c) and 500/1 (d–f), BPA as the initiator, Cu(0) and Cu^II^ as catalysts, and Me_6_TREN as the ligand.

To further investigate the potential of the above kinetically controlled strategy to obtain PAM with higher *M*_w_s, we applied it to the polymerization of aqueous Cu(0)-mediated RDRP of AM with [M]_0_/[I]_0_ = 200/1 and 500/1, respectively. By maintaining the optimized reaction parameters and feeding ratios above [M]:[Cu^II^]:[L] = 100/0.4/0.6, polymerization with [M]_0_/[I]_0_ – 200/1 was conducted by simply halving the amount of initiators. Once again, under good kinetic control, polymerization was well controlled with a monomer conversion over 90% in 90 min (entries 1–5 in Table 3, and Figure 3a–c). Specifically, the linear increase of *M*_n,NMR_ with conversion was clearly observed in Figure 3b; moreover, the number-average *M*_w_s matched well with the theoretical ones (e.g., *M*_n,th_ = 10.02 kDa, *M*_n,NMR_ = 9.53 kDa, entry 3 in Table 3), indicating good living polymerization. The well-controlled propagation process can be reflected by the parallel evolution of SEC traces (Figure 3c) and the narrow MWDs (low *Đ* ∼ 1.15) (Figure 3b).

**Table 3:** ^1^H NMR and SEC Analysis of Cu(0)-Mediated RDRP of AM with Varied *M*_w_s

entry	[M]:[I]:[Cu^II^]:[L]^a^	time (min)	*M*_n,th_^b^ (kDa)	*M*_n,NMR_^c^ (kDa)	*Đ*^d^	conv.^c^ (%)
1	200:1:0.8:1.2	10	2.82	2.72	1.13	19.9
2		20	7.65	7.24	1.16	53.8
3		30	10.02	9.53	1.13	70.5
4		45	11.58	11.75	1.13	81.5
5		90	13.13	12.90	1.14	92.4
6	500:1:2:3	10	11.11	11.08	1.17	31.3
7		20	20.73	20.35	1.14	58.3
8		30	24.11	23.20	1.13	67.8
9		90	32.88	31.95	1.14	92.5

a Reaction conditions: M = AM, I = BPA, Cu(0) = pretreated Cu(0) wire (*l* = 5 cm, *d* = 1 mm), Cu^II^ = CuBr_2_, L = Me_6_TREN, Solvent = H_2_O (20 mL), *T* = 25 °C, [M]_0_ = 1.5 M.

b *M*_n,th_ = ([M]_0_/[I]_0_) × Monomer conversion (Conv.) × *M*_w_ (M).

c Number-average molecular weights (*M*_n,NMR_) and Conv. were determined by ^1^H NMR (Figures S5 and S6).

d Dispersity (*Đ*) was characterized using size exclusion chromatography (SEC) equipped with an RI detector.

Furthermore, the aqueous Cu(0)-mediated RDRP of AM with [M]_0_/[I]_0_ of 500/1 was also carried out by maintaining the optimized reaction conditions but lowering the amount of initiators fivefold. Similarly, in this case, a well-controlled polymerization process was also achieved while retaining high monomer conversion (entries 6–9 in Table 3, and Figure 3d–f). In detail, the good livingness was represented by the linear increase of *M*_n,NMR_ with conversion (Figure 3e) and the expected *M*_w_s (^1^H NMR *M*_w_s in Table 3). In addition, the obvious parallel movements of MWDs with low dispersity (*Đ* < 1.15) were observed (Figure 3e,f). These results show that this kinetically controlled strategy overcame the long-standing challenges with the Cu-catalyzed polymerization of AM (low monomer conversion, poorly controlled *M*_w_s/MWDs, complex or multireaction procedures, etc.), allowing the successful synthesis of PAMs with a well-defined structure of a range of *M*_w_s.

## Conclusions

To conclude, the controlled polymerizations of acrylamide have been successfully achieved via a one-pot and one-step aqueous Cu(0)-mediated RDRP approach. It has been demonstrated that strong deactivation control is the key for the control in AM RDRPs. For the fast-propagating monomer AM, enhanced deactivation control can be achieved by the extra addition of Cu^II^ into the reaction system. This strategy overcame the long-standing challenges with the Cu-catalyzed polymerization of AM (low monomer conversion, poorly controlled *M*_w_s/MWDs, complex or multireaction procedures, etc.), allowing the successful synthesis of well-defined PAM with narrow MWDs and high monomer conversion of a range of *M*_w_s.

## Supporting Information Available

Materials, characterization methods, and experimental procedures; SEC data for PAM synthesized using only Cu(0) wire and Cu(0) wire and 15% Cu^II^ as the catalyst; chain extension results; SEC data and ^1^H NMR spectra for block copolymerization; PAM *M*_w_s calculated by SEC equipped with an RI detector and ^1^H NMR; and ^1^H NMR spectra for the PAM structure at various time intervals (PDF)
