# Supplementary material for: Controlled Polymerization of Acrylamide via One-Pot and One-Step Aqueous Cu(0)-Mediated Reversible-Deactivation Radical Polymerization
Source: Macromolecules. 2023 Jun 19;56(13):5111–6. doi: 10.1021/acs.macromol.3c00343 (PMC10339782; doi:10.1021/acs.macromol.3c00343)
Supplement: Supplementary file 1 — ma3c00343_si_001.pdf [file ma3c00343_si_001.pdf]

Supporting Information

Controlled Polymerization of Acrylamide *via* a One-pot and One-step Aqueous Cu(0)-Mediated Reversible-Deactivation Radical Polymerization

Zishan Li[2], Jing Lyu[,2], Yinghao Li[2], Bei Qiu[2], Melissa Johnson[2], Hongyun Tai[3], Wenxin Wang[*,1,2,4]*

[1] Aust Hefei Institute for Advanced Research, Anhui University of Science and Technology, Hefei, 232001, China

[2] Charles Institute of Dermatology, School of Medicine, University College Dublin, Dublin 4, D04 V1W8, Ireland

[3] School of Chemistry, Bangor University, Deiniol Road, Bangor, Gwynedd LL57 2UW, UK

[4] School of Mechanical and Materials Engineering, University College Dublin, Dublin 4, D04 V1W8, Ireland

*Corresponding author (email: wenxin.wang@ucd.ie, jing.lyu@ucd.ie)

METHODS AND MATERIALS

Materials

Acrylamide (AM, 99%, Aldrich), 2-hydroxyethyl acrylamide (HEAA, 97%, Aldrich), 2-Bromopropionamide (BPA, 99%, Aldrich), copper(II) bromide ($CuBr_2$, 99%, Aldrich), tris[2-(dimethylamino)ethyl]amine (Me_6TREN, 97%, Aldrich) were used as received. Copper wire was purchased from Sigma-Aldrich, then immersed in conc. HCl and rinsed with acetone and water before use. Water (from Siemens Ultra-Clear TWF Water Purification Systems) and Al_2O_3 (basic, Fisher) was used as received. Solvents were purchased from Fisher Scientific and used as received.

Characterization Methods

Number-average molecular weight ($M_{n,SEC}$), Weight-average molecular weight ($M_{w,SEC}$) and dispersity ($Đ$) were obtained by size exclusion chromatography (SEC) (Agilent GPC/SEC 50) equipped with a refractive index (RI) detector. The columns were eluted using water (30 cm PLgel Mixed-C * 2) and calibrated using a series of 12 near-monodisperse 6 PEG standards (M_p from 1.06×10^2 g/mol to 1.608×10^6 g/mol). The methods were set at a rate of 1 mL/min. ^{1}H Nuclear magnetic resonance spectroscopy (^{1}H NMR) analysis was carried out on a Varian NMR system 400 MHz spectrometer with MestRenova 6.1 processing software and reported in parts per million (ppm) relative to the response of D_2O (4.79 ppm) and tetramethylsilane (0.00 ppm).

Experimental Procedures

Polymerization procedure of AM *via* one-pot and one-step aqueous Cu(0)-mediated RDRP

The polymerizations were carried out in a two-necked round-bottom flask, with the

ratios of monomer and initiator set as 100/1, 200/1 and 500/1. For the ratio of 100/1, AM (2.13 g, 30 mmol, 100 equiv.), BPA (45.60 mg, 0.3 mmol, 1 equiv.), Me_6TREN (48.11 μL, 0.18 mmol, 0.6 equiv.), $CuBr_2$ (26.80 mg, 0.12 mmol, 0.4 equiv.) and H_2O (20 mL) were added into the two-neck flask and bubbled with argon to remove oxygen for 15 min. A stirrer bar wrapped with 5 cm of copper wire was immersed in conc. HCl and then thoroughly rinsed with acetone and water. After the pre-treated Cu(0) wire was dried, it was added to the flask quickly under a positive pressure of argon. The reaction was stirred at 600 r/min in an oil bath at ambient temperature, and polymerization was conducted for a desired period of reaction time. For the ratio of 200/1 and 500/1, the same polymerization procedures were used.

Purification of PAM polymers

The polymerization reactions were terminated by opening the sample vial, removing the Cu(0) wire, and exposing the catalyst and active radicals to air. The solution was then precipitated into a large excess of methanol. The copper ions were removed by filtering the polymer solution through an aluminium oxide column. The precipitated mixture was then dried under vacuum overnight at 65 °C and collected for further analysis.

Chain extension procedure of PAM-Br *via* one-pot and one-step aqueous Cu(0)-mediated RDRP

The PAM polymer generated at 90 mins from Cu(0)-mediated RDRP with the ratio of monomer and initiator of 100/1 (Table 2 in the main text) was purified following the above procedure as a macroinitiator. The chain extension polymerization was carried out by charging AM (0.39 g, 5.492 mmol, 200 equiv.), PAM-Br macroinitiator (173.40 mg, 0.027 mmol, 1 equiv.), Me_6TREN (17.61 μL, 0.066 mmol, 2.4 equiv.), $CuBr_2$ (9.81

mg, 0.044 mmol, 1.6 equiv.) and H_2O (10 mL) into the two-neck flask and bubbled with argon to remove oxygen for 15 min. A stirrer bar wrapped with 5 cm of copper wire was immersed in conc. HCl and then thoroughly rinsed with acetone and water. After the pre-treated Cu(0) wire was dried, it was added to the flask quickly under a positive pressure of argon. The reaction was stirred at 600 r/min in an oil bath at 60 °C, and polymerization was conducted for a desired period of reaction time.

Block copolymerization procedure of PAM-Br and HEAA *via* one-pot and one-step aqueous Cu(0)-mediated RDRP

The PAM polymer generated at 90 mins from Cu(0)-mediated RDRP with the ratio of monomer and initiator of 100/1 (under the recipe detailed in Table 2 in the main text) was purified following the above procedure as a macroinitiator. The block copolymerization was carried out by charging HEAA (0.28 g, 2.401 mmol, 100 equiv.), PAM-Br macroinitiator (140.00 mg, 0.024 mmol, 1 equiv.), Me_6TREN (46.20 μL, 0.173 mmol, 7.2 equiv.), $CuBr_2$ (25.74 mg, 0.115 mmol, 4.8 equiv.) and H_2O (10 mL) into the two-neck flask and bubbled with argon to remove oxygen for 15 min. A stirrer bar wrapped with 5 cm of copper wire was immersed in conc. HCl and then thoroughly rinsed with acetone and water. After the pre-treated Cu(0) wire was dried, it was added to the flask quickly under a positive pressure of argon. The reaction was stirred at 600 r/min in an oil bath at 60 °C, and polymerization was conducted for a desired period of reaction time.

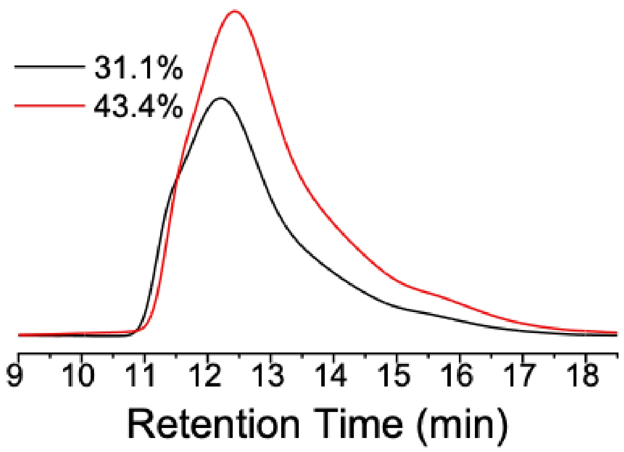

Figure S1. SEC traces of Cu(0)/Me_6TREN catalyzed polymerization of AM with BPA as initiator in H_2O at 25°C ($[M]_0/[I]_0$=100/1).

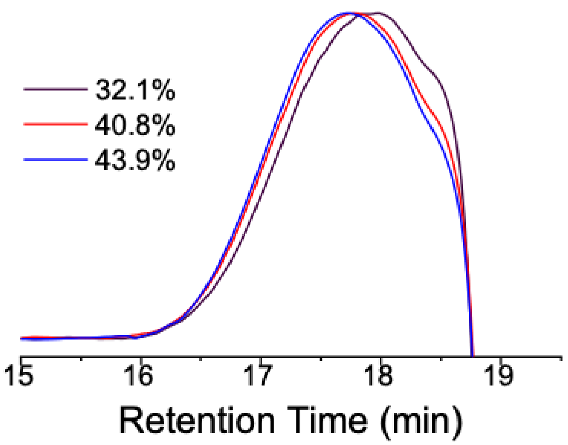

Figure S2. SEC traces of Cu(0)&15% Cu^{II}/Me_6TREN catalyzed polymerization of AM with BPA as initiator in H_2O at 25°C ($[M]_0/[I]_0$=100/1).

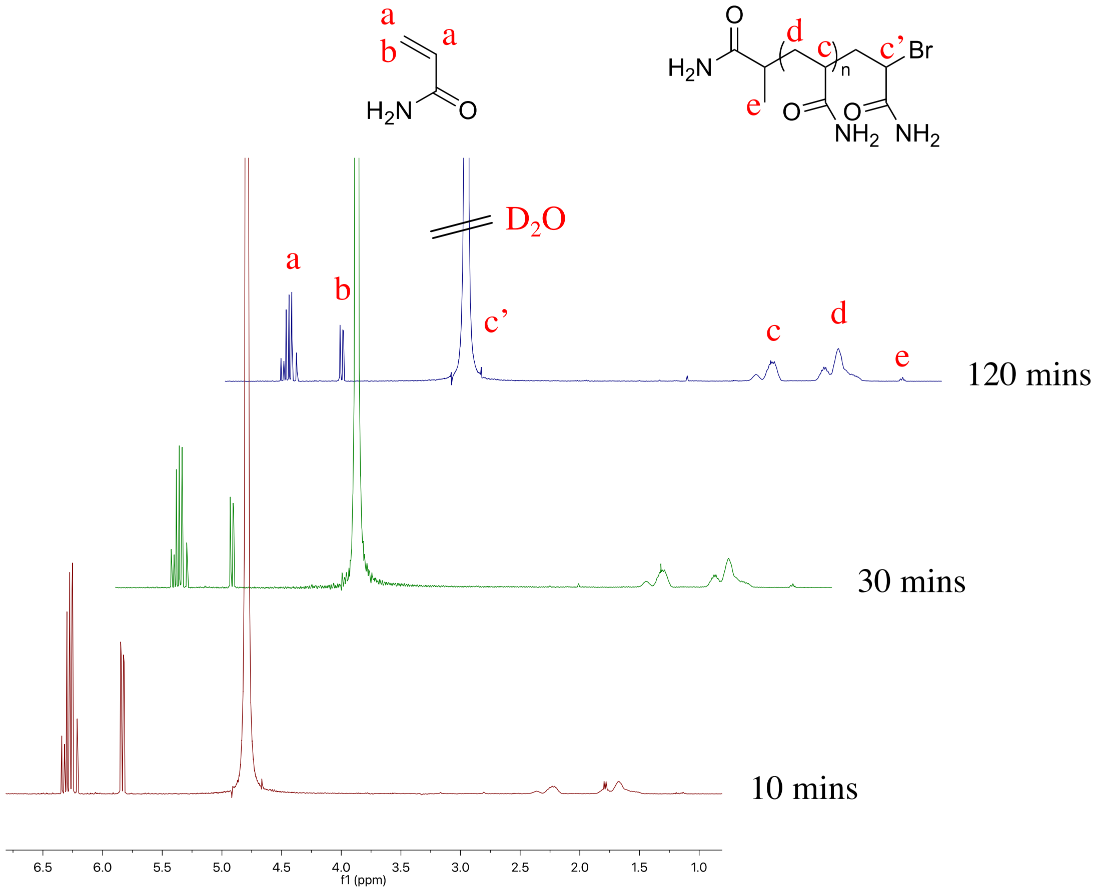

Figure S3. ^{1}H NMR spectra of PAM in D_2O with $[M]_0/[I]_0$ of 100/1 and 15% Cu^{II}.

Monomer conversion was calculated from ^{1}H NMR $=1-\frac{(a+b)/3}{(a+b)/3+(c+d)/3}=1-\frac{(a+b)}{(a+b)+(c+d)}$

Molecular weight calculated from ^{1}H NMR $M_{n,NMR}=\frac{d/2}{e/3}\times 71.08$

a+b = the integrals of monomer peak, c+d = the integrals of polymer peak, e = the integrals of chain end peak

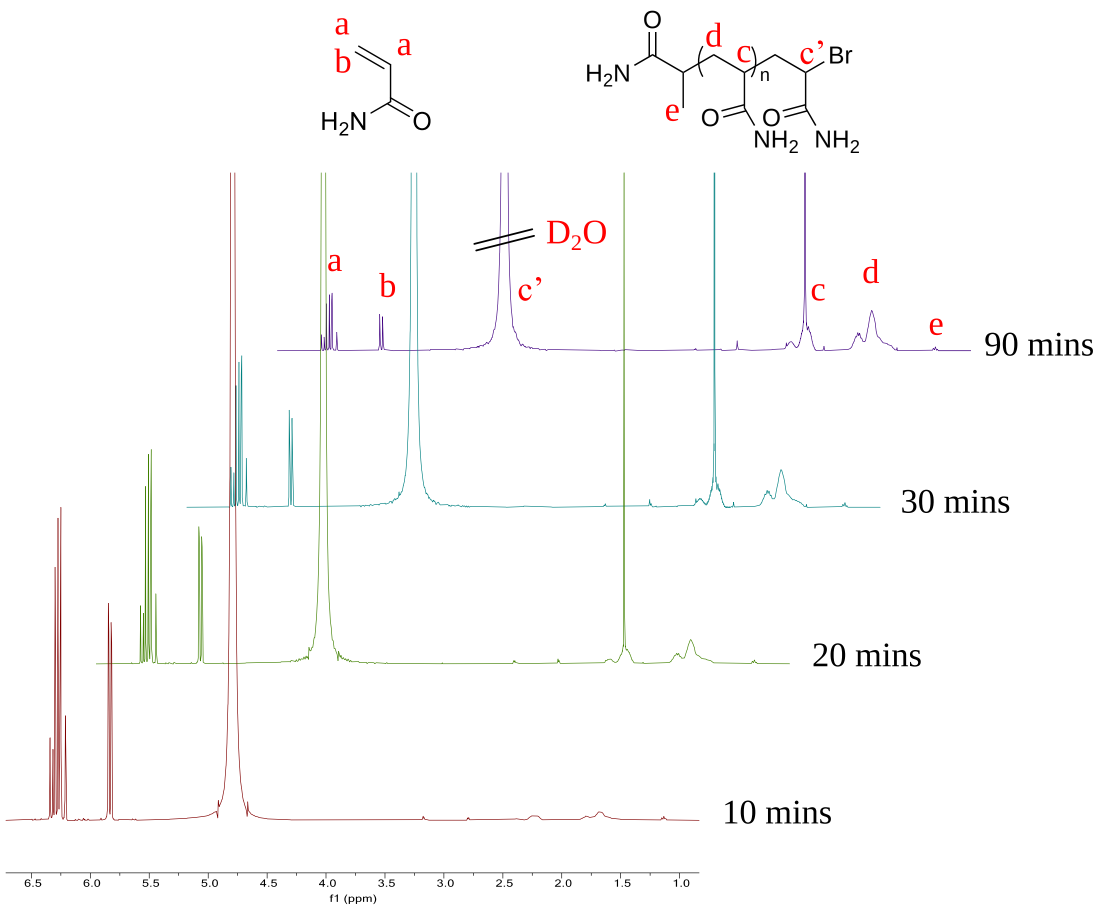

Figure S4. ^{1}H NMR spectra of PAM in D_2O with $[M]_0/[I]_0$ of 100/1 and 40% Cu^{II}.

Monomer conversion was calculated from ^{1}H NMR $=1-\frac{(a+b)/3}{(a+b)/3+(c+d)/3}=1-\frac{(a+b)}{(a+b)+(c+d)}$

Molecular weight calculated from ^{1}H NMR $M_{n,NMR}=\frac{d/2}{e/3}\times 71.08$

a+b = the integrals of monomer peak, c+d = the integrals of polymer peak, e = the integrals of chain end peak

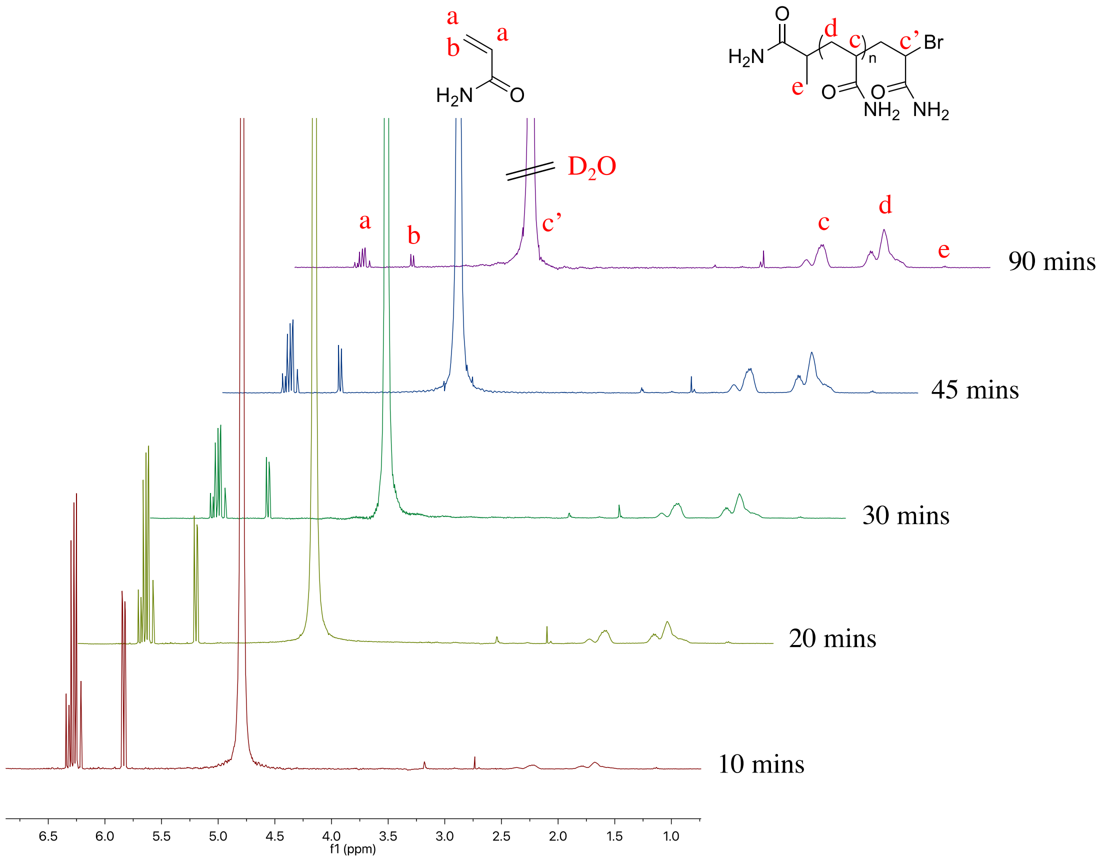

Figure S5. ^{1}H NMR spectra of PAM in D_2O with $[M]_0/[I]_0$ of 200/1.

Monomer conversion was calculated from ^{1}H NMR $=1-\frac{(a+b)/3}{(a+b)/3+(c+d)/3}=1-\frac{(a+b)}{(a+b)+(c+d)}$

Molecular weight calculated from ^{1}H NMR $M_{n,NMR}=\frac{d/2}{e/3}\times 71.08$

a+b = the integrals of monomer peak, c+d = the integrals of polymer peak, e = the integrals of chain end peak

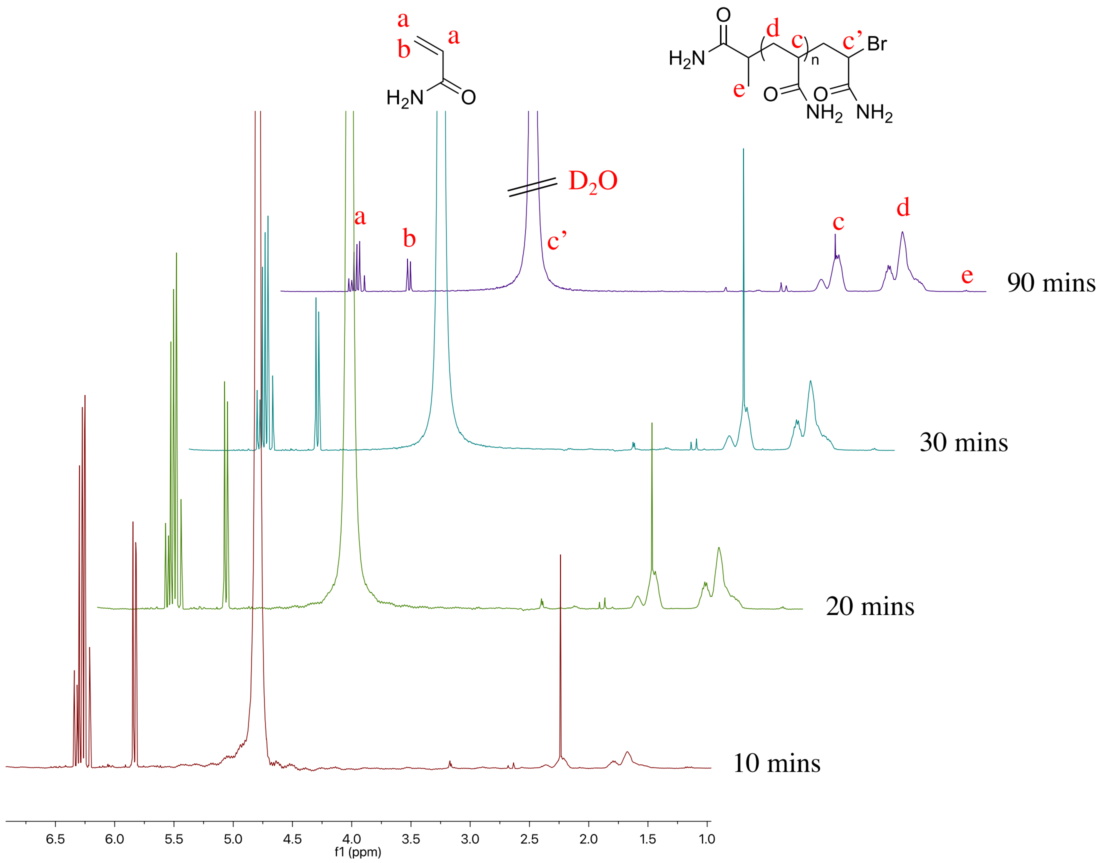

Figure S6. ^{1}H NMR spectra of PAM in D_2O with $[M]_0/[I]_0$ of 500/1.

Monomer conversion was calculated from ^{1}H NMR $=1-\frac{(a+b)/3}{(a+b)/3+(c+d)/3}=1-\frac{(a+b)}{(a+b)+(c+d)}$

Molecular weight calculated from ^{1}H NMR $M_{n,NMR}=\frac{d/2}{e/3}\times 71.08$

a+b = the integrals of monomer peak, c+d = the integrals of polymer peak, e = the integrals of chain end peak

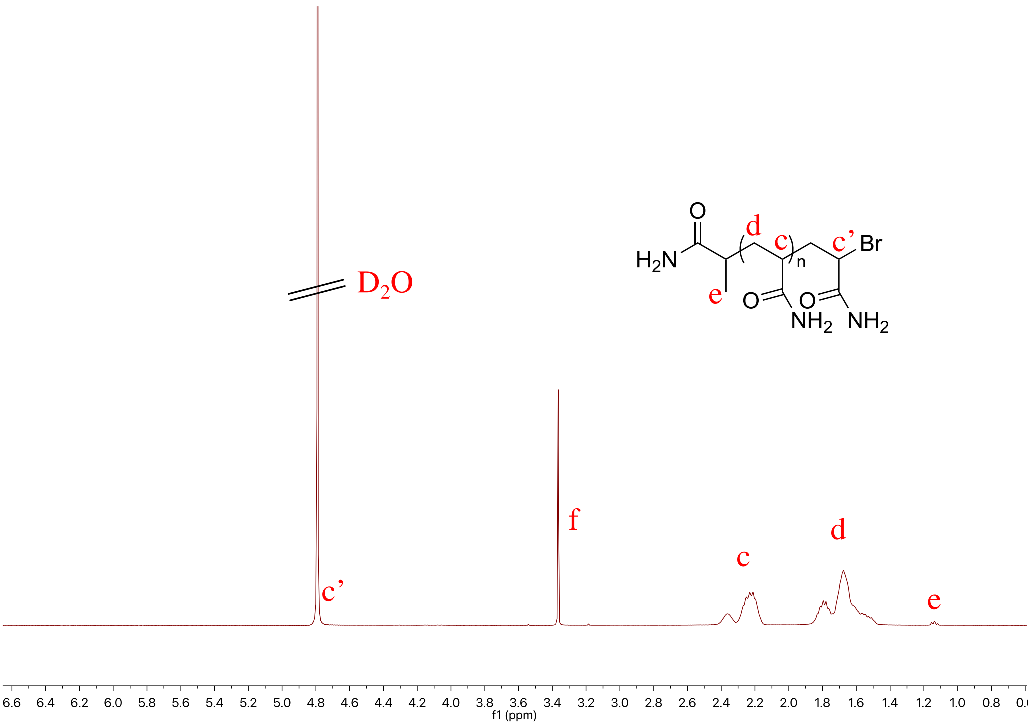

Figure S7. ^{1}H NMR spectra of purified macroinitiator (PAM-Br) in D_2O. The macroinitiator was synthesized from Cu(0)-mediated RDRP of AM with $[M]_0/[I]_0$ of 100/1 and 40% Cu^{II} (Entry 4, Table 2).

Molecular weight calculated from ^{1}H NMR $M_{n,NMR} = \frac{d/2}{e/3} \times 71.08$

c+d = the integrals of polymer peak, e = the integrals of chain end peak

f = residual methanol

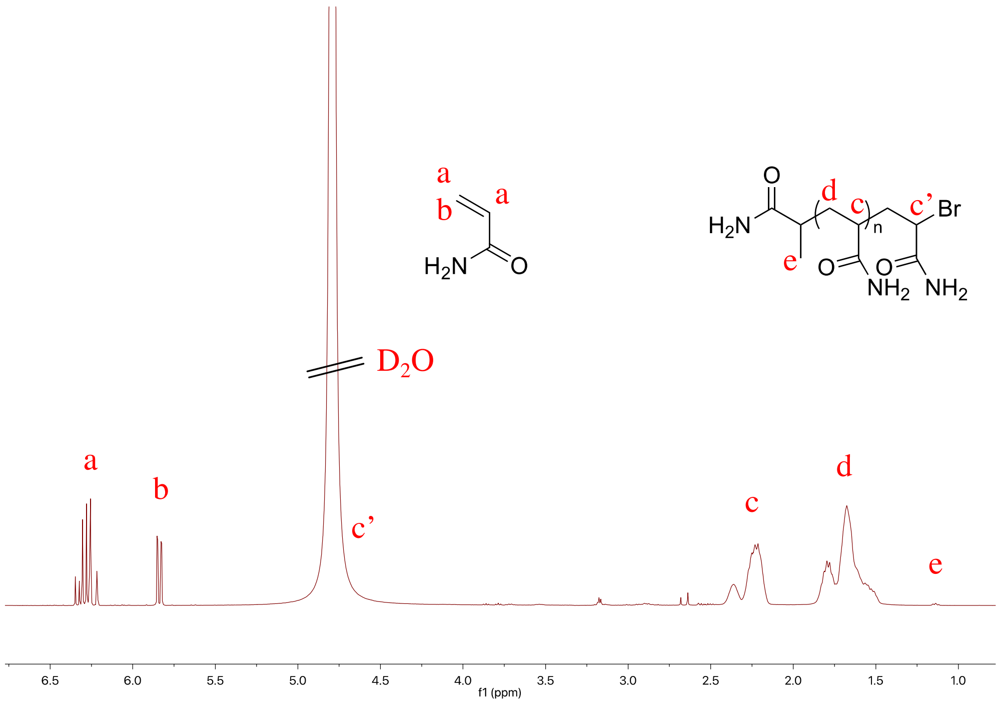

Figure S8. ^{1}H NMR spectra of chain extended polymer (PAM-Br-PAM) in D_2O after 6 days chain extension polymerization period with $[M]_0/[PAM\text{-}Br]_0$ of 200/1.

Molecular weight calculated from ^{1}H NMR $M_{n,NMR} = \frac{d/2}{e/3} \times 71.08$

Monomer conversion was calculated from ^{1}H NMR

$$= \frac{\frac{d_{PAM-Br-PAM}/2}{e_{PAM-Br-PAM}/3} \times 71.08 - \frac{d_{PAM-Br}/2}{e_{PAM-Br}/3} \times 71.08}{71.08 \times 200} = \frac{3 \times \left(\frac{d_{PAM-Br-PAM}}{e_{PAM-Br-PAM}} - \frac{d_{PAM-Br}}{e_{PAM-Br}}\right)}{400}$$

a+b = the integrals of monomer peak, c+d = the integrals of polymer peak, e = the integrals of chain end peak

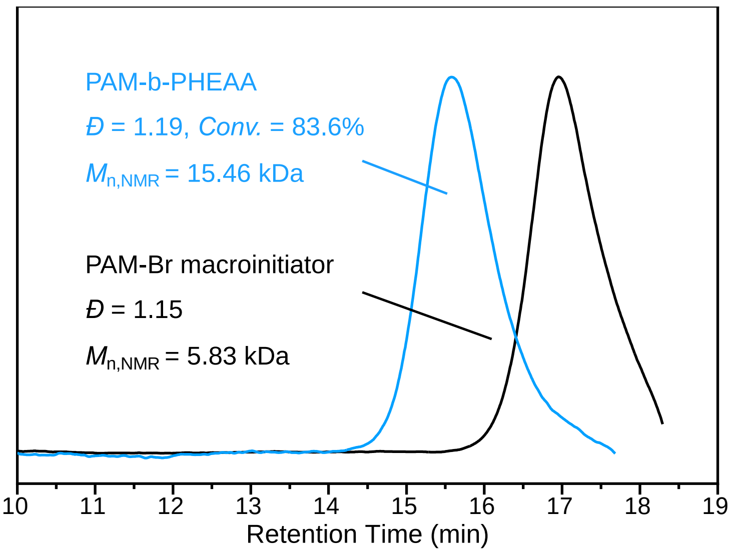

Figure S9. SEC traces of the PAM-Br macroinitiator ($Đ$ = 1.15, $M_{n,NMR}$ = 5.83 kDa) and block copolymer PAM-b-PHEAA ($Đ$ = 1.19, $M_{n,NMR}$ = 15.46 kDa) synthesized *via* one-pot and one-step aqueous Cu(0)-mediated RDRP.

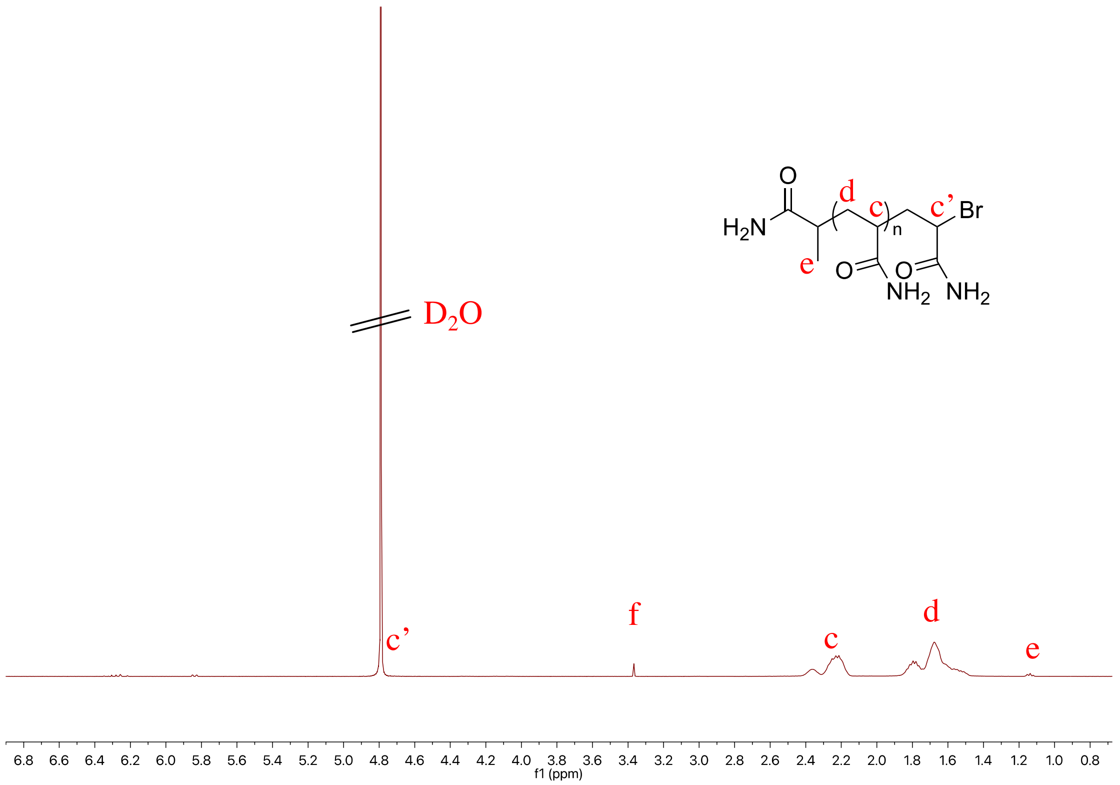

Figure S10. ^{1}H NMR spectra of purified macroinitiator (PAM-Br) in D_2O. The macroinitiator was synthesized from Cu(0)-mediated RDRP of AM with $[M]_0/[I]_0$ of 100/1 and 40% Cu^{II} (under the same recipe as described in Table 2).

Molecular weight calculated from ^{1}H NMR $M_{n,NMR} = \frac{d/2}{e/3} \times 71.08$

c+d = the integrals of polymer peak, e = the integrals of chain end peak

f = residual methanol

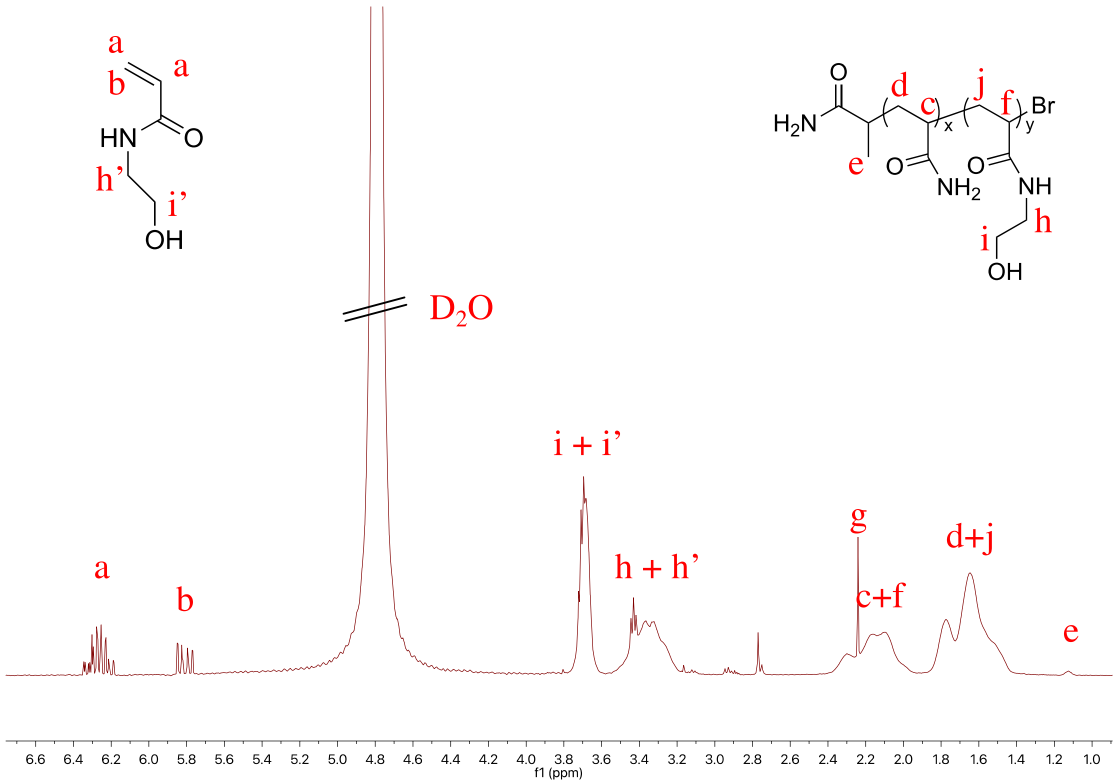

Figure S11. ^{1}H NMR spectra of block copolymer (PAM-b-PHEAA) in D_2O with $[M]_0/[PAM\text{-}Br]_0$ of 100/1.

Molecular weight calculated from ^{1}H NMR $M_{n,NMR}$

$$=\frac{d_{PAM-Br}/2}{e_{PAM-Br}/3}\times 71.08+\left(\frac{d+j_{PAM-b-PHEAA}/2}{e_{PAM-b-PHEAA}/3}-\frac{d_{PAM-Br}/2}{e_{PAM-Br}/3}\right)\times 115.13$$

Monomer conversion was calculated from ^{1}H NMR

$$=\frac{\frac{d+j_{PAM-b-PHEAA}/2}{e_{PAM-b-PHEAA}/3}-\frac{d_{PAM-Br}/2}{e_{PAM-Br}/3}}{100}$$

a+b, i'+h' = the integrals of monomer peak, c+f, d+j, i and h = the integrals of polymer peak, e = the integrals of chain end peak, g = residual acetone

Table S1. ^{1}H NMR characterization results of Cu(0)-mediated RDRP of AM with Cu(0)&15% Cu^{II} as catalyst

Entry	[M]:[I]:[Cu^{II}]:[L][a]	Time (min)	$M_{n,th}$[b] (kDa)	$M_{n,NMR}$[c] (kDa)	*Conv.*[c] (%)
1	100:1:0.15:0.23	10	2.45	2.83	34.4
2		30	3.09	3.32	43.5
3		120	3.28	3.49	46.2

[a]Reaction conditions: M = AM, I = BPA, Cu(0) = pre-treated Cu(0) wire (l = 5 cm, d = 1 mm), Cu^{II} = $CuBr_2$, L = Me_6TREN, Solvent = H_2O (20 mL), T = 25 °C, $[M]_0$ = 1.5 M; [b]$M_{n,th}$ = ($[M]_0/[I]_0$) × Monomer conversion (*Conv.*) × MW (M); [c]Number-average molecular weights ($M_{n,NMR}$) and *Conv.* was determined by ^{1}H NMR (Figure S3).

Table S2. ^{1}H NMR and SEC characterization results of Cu(0)-mediated RDRP of AM with varied MWs

Entry	[M]:[I]:[Cu^{II}]:[L][a]	Time (min)	Molar ratio of polymer to chain end[b]	$M_{n,th}$[c] (kDa)	$M_{n,NMR}$[d] (kDa)	$M_{n,SEC}$[e] (kDa)	*Conv.*[d] (%)
1	100:1:0.4:0.6	10	18/1	1.25	1.30	2.10	17.6
2		20	53/1	3.71	3.75	2.80	52.2
3		30	69/1	4.85	4.91	3.51	68.3
4		90	88/1	6.18	6.24	4.68	86.9
5	200:1:0.8:1.2	10	19/1	2.82	2.72	2.63	19.9
6		20	51/1	7.65	7.24	8.17	53.8
7		30	67/1	10.02	9.53	10.25	70.5
8		45	83/1	11.58	11.75	11.65	81.5
9		90	91/1	13.13	12.90	14.07	92.4
10	500:1:2:3	10	31/1	11.11	11.08	10.45	31.3
11		20	57/1	20.73	20.35	20.27	58.3
12		30	65/1	24.11	23.20	23.67	67.8
13		90	90/1	32.88	31.95	31.92	92.5

[a]Reaction conditions: M = AM, I = BPA, Cu(0) = pre-treated Cu(0) wire (l = 5 cm, d = 1 mm), Cu^{II} = $CuBr_2$, L = Me_6TREN, Solvent = H_2O (20 mL), T = 25 °C, $[M]_0$ = 1.5 M; [b]Molar ratio of polymer to chain end was measured by ^{1}H NMR (polymer/chain end = (d/2)/(e/3), Figure S4, Figure S5 and Figure S6); [c]$M_{n,th}$ = ($[M]_0/[I]_0$) × Monomer conversion (*Conv.*) × MW (M); [d]Number-average molecular weights ($M_{n,NMR}$) and *Conv.* was determined by ^{1}H NMR (Figure S4, Figure S5 and Figure S6). [e]Number-average molecular weights ($M_{n,SEC}$) was characterized using size exclusion

chromatography (SEC) equipped with an RI detector.
